# Photocatalytic degradation of ofloxacin in water assisted by TiO_2_ nanowires on carbon cloth: contributions of H_2_O_2_ addition and substrate absorbability

**DOI:** 10.3762/bjnano.16.111

**Published:** 2025-09-08

**Authors:** Iram Hussain, Lisha Zhang, Zhizhen Ye, Jin-Ming Wu

**Affiliations:** 1 State Key Laboratory of Silicon and Advanced Semiconductor Materials, School of Materials Science and Engineering, Zhejiang University, Hangzhou, 310027, P. R. China; 2 Zhejiang Provincial Engineering Research Center of Oxide Semiconductors for Environmental and Optoelectronic Applications, Institute of Wenzhou, Zhejiang University, Wenzhou 325006, P. R. China

**Keywords:** carbon cloth, ofloxacin, photocatalytic degradation, TiO_2_ nanowires, wastewater treatment

## Abstract

Vertically aligned TiO_2_ nanowires demonstrate exceptional photoactivity owing to their high specific surface area and improved charge separation; however, their unsatisfactory interaction with target contaminants diminishes photocatalytic degradation efficiency in water. Here, we present a mild solution method to precipitate anatase TiO_2_ nanowire arrays, measuring 1.5 μm in thickness, over carbon cloth to ensure substantial interactions with target pollutants and, in turn, a superior photoactivity. Compared to TiO_2_ nanowire arrays grown on metallic Ti substrates, TiO_2_ nanowires supported on carbon cloth substrates demonstrate markedly superior efficiency in the photocatalytic degradation of ofloxacin (OFL) molecules in water when exposed to UV light. The TiO_2_ nanowires remove 90–97% OFL in water with a high initial concentration of 50 ppm in 6 h under UV light irradiation for up to six cycles. The contributions of the hydrogen peroxide (H_2_O_2_) additive were also studied. An enhanced efficiency could be achieved only when the H_2_O_2_ in water reaches a critical amount, below which a negative effect is noted. This investigation demonstrates the potential of improving the photoactivity of one-dimensional TiO_2_ nanostructures by utilizing a highly adsorptive substrate, which can help mitigate the effects of hazardous materials in water.

## Introduction

Drug pollution in natural environments has become an increasingly serious issue as a result of the wide applications of drugs in the diagnosis and treatment of human diseases, anti-epidemic disinfection, aquaculture additives, and veterinary drugs [1–2]. Ofloxacin (OFL) is a broad-spectrum fluoroquinolone antibiotic widely used in clinical treatments for various bacterial infections [3]. After administration, OFL is not fully metabolized and is primarily excreted through urine, leading to its presence in wastewater and contamination of aquatic environments. Studies have reported OFL concentrations in surface waters ranging from 0.05 to 17.7 μg/L, posing a potential risk to aquatic organisms and disrupting ecosystem balance [4–5]. Therefore, OFL removal from water is an important issue in environmental science and engineering.

Conventional treatment approaches, including adsorption, membrane separation, and biological methods, are largely inadequate for antibiotics such as OFL [6–7]. This limitation highlights the need for advanced oxidation processes, with a particular emphasis on TiO_2_-based photocatalysis [8]. However, TiO_2_ as a sole component presents challenges partly due to the rapid recombination of photogenerated electron–hole pairs, which significantly reduces its photocatalytic efficiency and practical applicability [9–10].

Recent advancements have focused on TiO_2_ photocatalysts supported on porous materials, such as carbon-based adsorbents. These innovations help to slow down electron–hole recombination, broaden light absorption, and enhance surface adsorption sites [11]. Cao et al. synthesized TiO_2_ nanowires on reduced graphene oxide (rGO) through a solvothermal method, which improved active sites and facilitated interfacial charge separation [12]. Activated carbon effectively supports TiO_2_, enhancing adsorption capacity and loading, which directly impacts photocatalytic efficiency [13]. In contrast to rGO or activated carbon granules, employing carbon cloth as substrates mitigates the aggregation issues and simplifies the separation and recovery of photocatalysts within a slurry system. TiO_2_ nanowires are known for their significantly high surface area and are important for photocatalytic and photoelectrochemical applications. Template-mediated methods such as sol–gel deposition, electrophoretic deposition, and electrochemical deposition are extensively utilized for the synthesis of TiO_2_ nanowire arrays, owing to their advantageous physical properties and varied applications [14–15].

Our previous study accomplished the precipitation of TiO_2_ nanowire arrays, with controlled film thickness of 1.5–4.5 μm, on concurrently activated carbon cloth substrates. The composite film exhibited a high efficiency towards removal of rhodamine B and sulfosalicylic acid in water under UV light illumination, mainly thanks to the highly adsorptive substrate [16]. In the current investigation, we extend the application to drug pollution treatments with OFL as a model molecule. Since H_2_O_2_ produces reactive oxygen species (ROS), which promote the oxidation and degradation of OFL, H_2_O_2_ was introduced into the system to increase the photocatalytic efficiency, and the impacts of its concentration were studied. The higher production of hydroxyl radicals (•OH) in the degradation of organic pollutants enhances the degradation efficiency of OFL. The TiO_2_ nanowires exhibited significant photodegradation of OFL in water with a high initial concentration range of 50–200 ppm, which is much higher than that of 10–20 ppm adopted by most studies, highlighting the advantages of the one-dimensional TiO_2_ material on an adsorptive substrate as a photocatalyst.

## Experimental

### Materials and reagents

Carbon cloth with a purity of 99.8% was provided by Shanghai Hesen Industry Company Limited, China. Hydrogen peroxide (30 wt %, AR), HNO_3_ (65 wt %, AR), and ethyl alcohol (99.7 wt %, AR) were purchased from Sinopharm Chemical Reagent Co., Ltd., China. Melamine (99 wt %, AR) was purchased from Aladdin, China. PHILPS Lighting Investment Company Limited, China, provided the UV lamp. All chemicals were used without further purification. Deionized water was used for all the experiments.

### Synthesis of TiO_2_ nanowires on carbon cloth

The synthesis follows basically our previous study but on a larger scale [16]. In brief, a 5 cm × 10 cm piece of carbon cloth (CC) underwent three cleaning cycles using ethyl alcohol and deionized water alternately in an ultrasonic bath, followed by drying at 60 °C. After that, it was immersed in 200 mL of a 30 wt % H_2_O_2_ solution, containing 60 mg of melamine, 4 mL of 65 wt % HNO_3_, and 1 g of titanium sponge, at 80 °C for 24 h to facilitate the precipitation of hydrogen titanate nanowires. Final calcination in air at 450 °C for 1 h was conducted to decompose titanates into anatase TiO_2_ and simultaneously activate the carbon cloth to achieve a high specific surface area [17]. The carbon cloth on which TiO_2_ nanowire arrays were precipitated is referred to as CC/NW-450°C. For comparisons, anatase TiO_2_ nanowire arrays on metallic Ti foils were synthesized according to previous reports [18] and designated as Ti/NW-450 °C. Commercial Degussa P25 TiO_2_ nanoparticles were also loaded on Ti substrates 5 × 10 × 0.01 cm^3^ in size by repetitive dip coating in an ethanolic P25 slurry and drying. The P25 loading was ca. 50.0 mg (≈1.0 mg/cm^2^), nearly the same as that of the TiO_2_ nanowires on carbon cloth.

### Characterizations

The morphologies were analyzed using field-emission scanning electron microscopy (FESEM, Zeiss Germany Sigma 300) and high-resolution transmission electron microscopy (HRTEM) conducted with a JEM-2100 microscope (Jeol, Tokyo, Japan). Energy-dispersive X-ray (EDX) elemental mapping was performed using the FESEM system to examine the distribution of Ti and O on the carbon cloth substrate. X-ray diffraction (XRD) measurements were conducted using a SmartLab diffractometer (Rigaku Corporation) with Cu Kα radiation, operating at 40 kV and 35 mA. Raman spectra were obtained using an Alpha300R UV system (WITec, Germany) equipped with a TEM00 laser at a wavelength of 532 nm. X-ray photoelectron spectroscopy (XPS) characterizations were conducted using a Kratos AXIS Ultura DLD system (Kratos, UK), and the binding energies were calibrated to C 1s = 284.8 eV. The specific surface area and pore size were analyzed using low-temperature N_2_ adsorption–desorption measurements conducted with an ASAP 2460 (USA). The absorption spectrum of the sample was acquired through UV–vis diffuse reflectance spectroscopy (UV-3150, Shimadzu, Japan). The concentration of OFL was determined using a Nexera LC-40D XR ultrahigh-performance liquid chromatography (HPLC) system (SHIMADZU, Japan). A Pyris 1 TGA instrument (USA) was used to carry out the thermogravimetric (TG) investigations of as-precipitated nanowires in air at a heating rate of 10 K·min^−1^ from room temperature to 500 °C. The static water contact angles were measured using a goniometer (OCA 11, DataPhysics Instruments GmbH, Filderstadt, Germany) following the standard sessile drop method. The measurements were performed at room temperature. A TOC-L CPH analyzer (SHIMADZU, Japan) was used to detect total organic carbon (TOC).

### Photocatalytic activity for OFL removal

The photocatalytic activity was evaluated through the photodegradation of 50 mL aqueous solutions of OFL at initial concentrations of 50, 75, 100, and 200 ppm, utilizing carbon cloth coated with nanowires with a nominal area of 5 cm × 10 cm in a Pyrex reactor. A UV lamp with a power of 18 W was positioned 5.5 cm above the fluid to provide irradiation. The average UV intensity at the solution level was approximately 5.0 mW·cm^−2^, measured using an irradiance meter (Model UV-A, Beijing Normal University). The solution was maintained in darkness, and the UV light was activated following the establishment of a dark adsorption–desorption equilibrium for three hours. The degradation experiments were repeated in triplicate to ensure statistical reliability. Error bars represent the standard deviation calculated from these repeated experiments.

The adsorption removal ratio of OFL was calculated using Equation 1:


[1] $$\text{Adsorption Removal Ratio }\left(\%\right)=\left(\frac{c_{0}-c_{\text{ads}}}{c_{0}}\right)\times 100\%,$$

where *c*_0_ represents the initial concentration of OFL (ppm), and *c*_ads_ denotes the concentration of OFL after the adsorption (ppm). The photodegradation ratio of OFL was determined using Equation 2:


[2] $$\text{Photodegradation Ratio }\left(\%\right)=\left(\frac{c_{\text{ads}}-c_{t}}{c_{\text{ads}}}\right)\times 100\%,$$

where, *c*_ads_ represents the concentration of OFL after the dark adsorption (ppm), while *c_t_* denotes the concentration of OFL at time *t* during the photodegradation (ppm). The reaction rate constant (*k*) of the OFL degradation was assessed using the pseudo-first-order kinetic model, as determined by Equation 3:


[3] $$\ln\left(\frac{c_{t}}{c_{\text{ads}}}\right)=-kt$$

The concentration of OFL was assessed using high-performance liquid chromatography with a C18 column maintained at 30 °C. The UV–vis detector was calibrated to a wavelength of 292 nm. The mobile phase consisted of a 15:85 ratio of methanol to 0.8% acetic acid in ultrapure water, with a flow rate set at 1.0 mL·min^−1^ and an injection volume of 10 μL. Every hour, 0.2 mL of OFL solution was sampled. The photocurrent was tested using a three-electrode system on an electrochemical workstation (CHI600E, Shanghai Chenhua), in which the carbon cloth (2 cm × 2 cm in size) was taken as working electrode, the Pt electrode as counter electrode (3 cm × 3 cm in size), and a saturated calomel electrode as reference electrode; the electrolyte is 0.5 mol/L aqueous Na_2_SO_4_ solution. No bias potential was applied for the photocurrent evaluations.

## Results and Discussion

### Microstructure and morphology of photocatalysts

**Figure 1:**
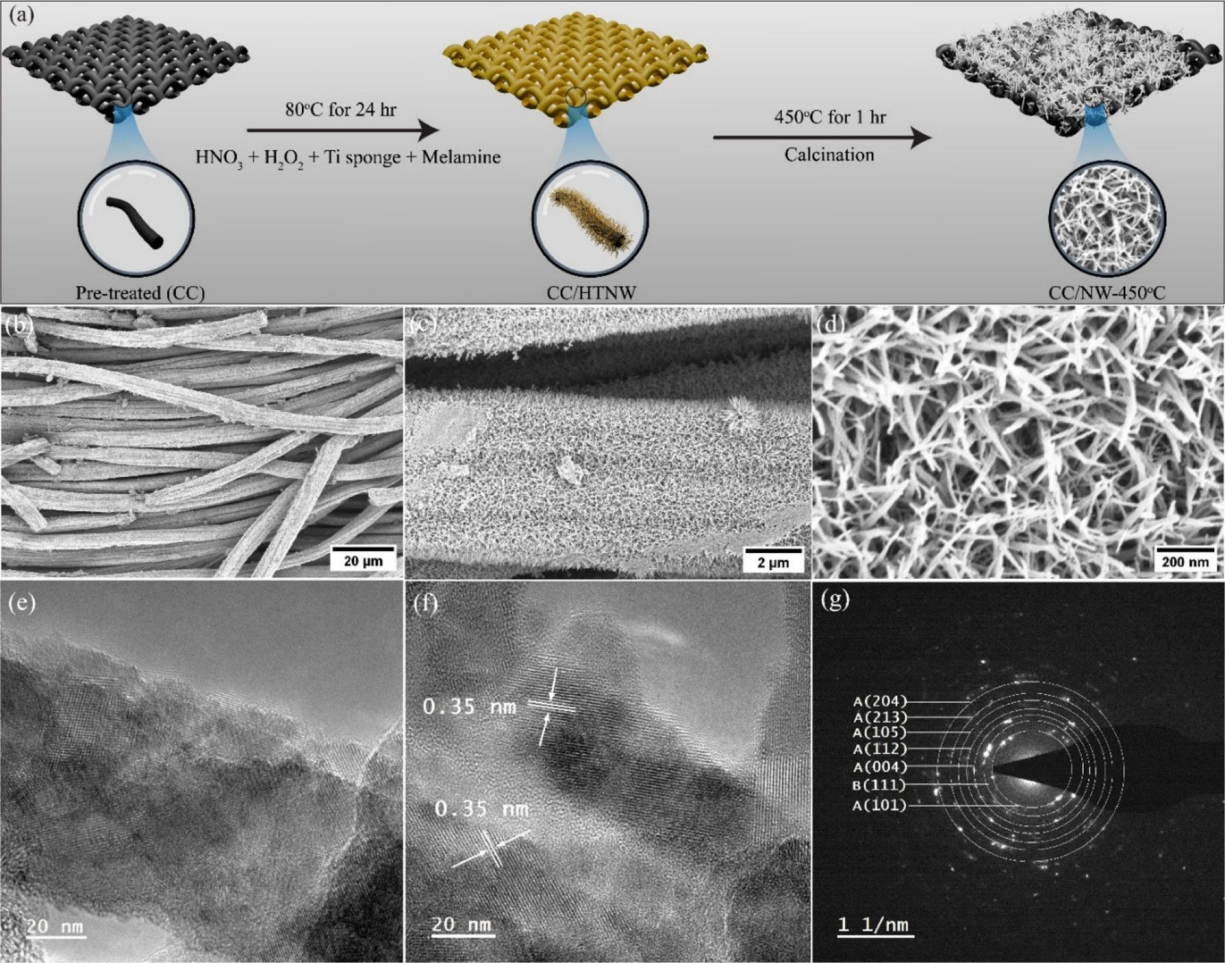
(a) The Ti–H_2_O_2_ interaction approach to precipitate TiO_2_ nanowire arrays on carbon cloth. (b–d) FESEM images of TiO_2_ nanowires grown on carbon cloth at different magnifications. (e) TEM, (f) HRTEM images, and (g) the corresponding SAED pattern of the CC/NW-450 °C.

Figure 1a shows schematically the synthesis of TiO_2_ nanowires on an activated carbon cloth substrate. When the carbon cloth is immersed in the Ti–H_2_O_2_ precursor solution maintained at 80 °C, first, the carbon cloth surface is activated, and, second, the hydrogen titanate nanowires precipitate. In the acidic medium, the reaction between metallic Ti sponge and hydrogen peroxide produces titanium hydroxide species in solution. Once the concentration of these species reaches saturation, hydrogen titanate nanowires nucleate and grow directly on the carbon cloth substrate via a heterogeneous nucleation mechanism [19–20]. Figure S1 in Supporting Information File 1 shows the homogeneous precipitation of hydrogen titanate nanowires on the carbon cloth. Subsequent calcination in air at 450 °C transformed the hydrogen titanate structures into anatase TiO_2_ nanowires, while largely retaining their original one-dimensional morphology. Figure 1b–d presents representative FESEM images of the resulting TiO_2_ nanowires uniformly deposited on the carbon cloth substrate. Each carbon fiber is evenly coated with quasi-aligned nanowires that radiate outward, forming a net-like architecture. After reacting in the Ti–H_2_O_2_ aqueous system at 80 °C for 24 h, the synthesized nanowire layer reaches an approximate thickness of 1.5 μm, covering the substrate homogeneously. Additionally, EDX mapping analysis (Supporting Information File 1, Figure S2) confirms that Ti and O are homogeneously distributed over the carbon cloth fibers, supporting the uniform and successful growth of TiO_2_ nanowires. The successful formation of TiO_2_ nanowires is further confirmed by TEM analysis. As shown in Figure 1e,f, the CC/NW-450 °C sample displays a distinct net-like network of nanowires. The corresponding selected-area electron diffraction (SAED) pattern reveals clear diffraction rings attributable to anatase and minor brookite TiO_2_ phases. Additionally, the HRTEM image (Figure 1f) shows well-defined lattice fringes with a spacing of approximately 0.35 nm, which corresponds to the (101) crystal plane of anatase TiO_2_.

**Figure 2:**
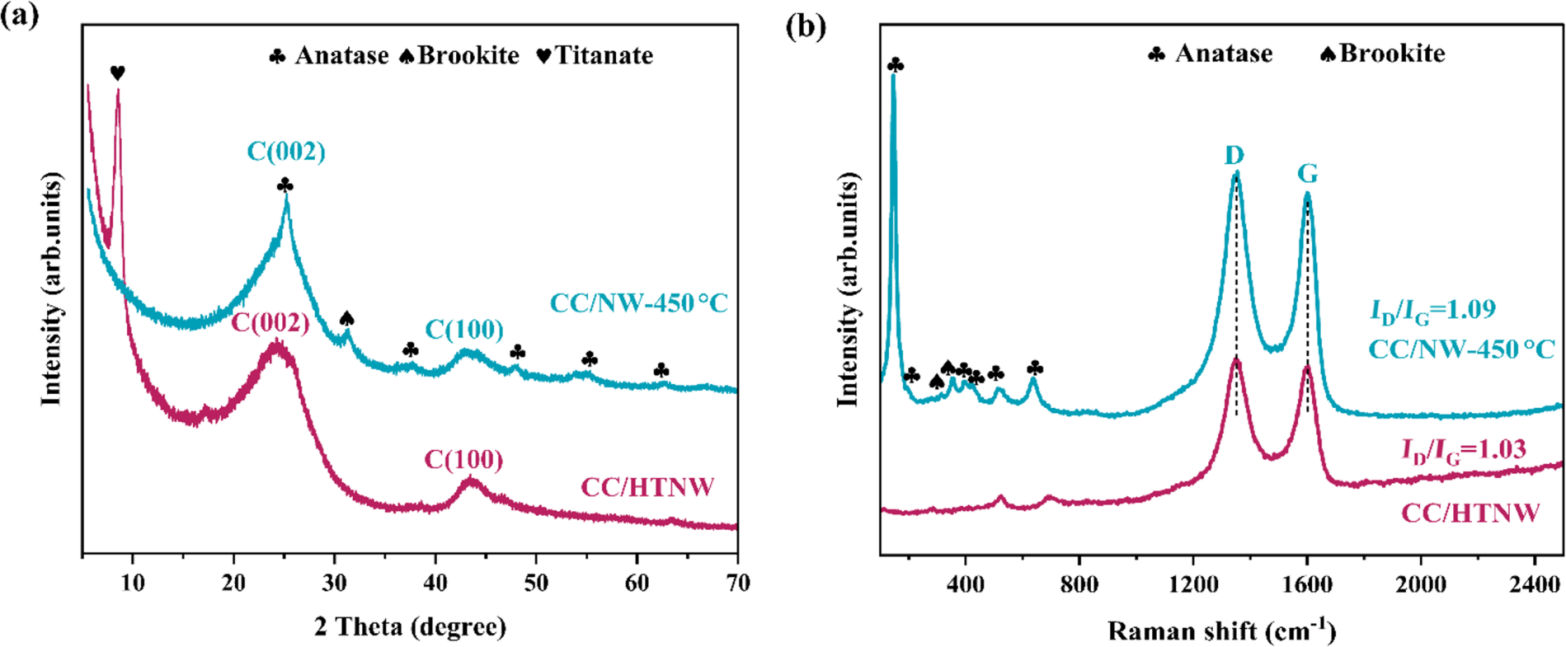
(a) XRD patterns and (b) Raman spectra of hydrogen titanate nanowires (CC/HTNW) and TiO_2_ nanowires (CC/NW-450 °C) grown on carbon cloth.

Figure 2a shows the XRD patterns of hydrogen titanate nanowires precipitated on carbon cloth (CC/HTNW) and of the sample after air calcination (CC/NW-450 °C). Except for two broad XRD peaks arising from the carbon substrate, a peak located at ca. 8.5° can be seen for the CC/HTNW, which corresponds to hydrogen titanate H_2_Ti_5_O_11_·3H_2_O (JCPDS card no. 44-0130). The TiO_2_ nanowires are predominantly in the anatase phase, as indicated by characteristic anatase peaks, such as the (101), (004), (200), and (105) reflections at 2θ = 25.3°, 37.8°, 48.0°, and 54.0°, respectively (JCPDS card no. 21-1272). The Raman spectra in Figure 2b are in accordance with the XRD analysis in phase composition. The CC/NW-450 °C spectrum displays prominent peaks at 146, 195, 287, 395, 516, and 637 cm^−1^, corresponding to the E_g_, A_1g_, B_1g_, B_1g,_ (A_1g_ + B_1g_), and E_g_ modes of the anatase phase, respectively [21–23]. Characteristic peaks corresponding to brookite TiO_2_ can also be seen in the XRD pattern and the Raman spectrum of CC/NW-450 °C. The Raman spectrum in Figure 2b also indicates the degree of disorder in the carbon cloth by the ratio (*I*_D_/*I*_G_) of the disorder-induced (D) band (≈1350 cm^−1^) and the graphite (G) band (≈1582 cm^−1^) [17]. The increasing ratio suggests an increasing surface disordering of the carbon cloth after calcination, which contributes to the high adsorption capacity toward the target molecules.

**Figure 3:**
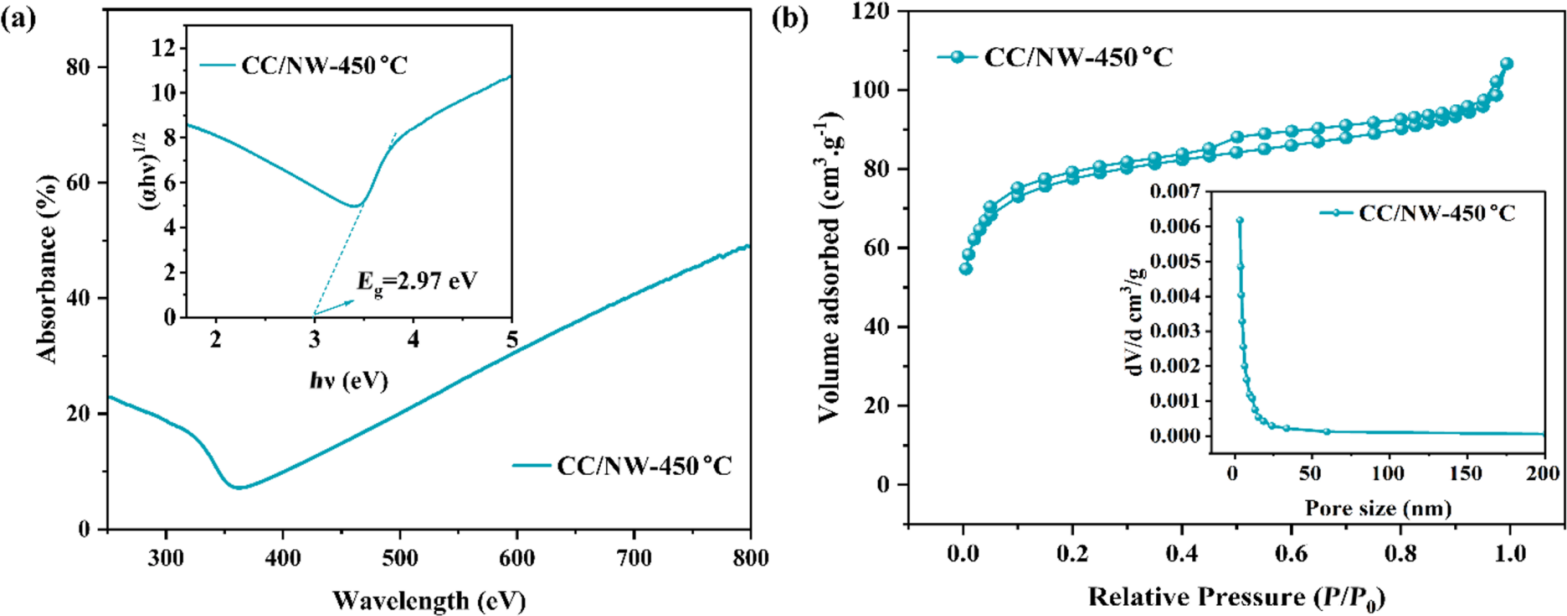
(a) UV–vis diffuse absorbance spectrum of the CC/NW-450 °C. The inset shows the replotting of (a) in (α*h*ν)^1/2^ ~ *h*ν coordinates to evaluate the bandgap of CC/NW-450 °C. (b) Low-temperature nitrogen adsorption–desorption isotherm of CC/NW-450 °C. The inset shows the corresponding pore size distribution curve.

Figure 3a illustrates the UV–vis diffuse absorbance spectrum of the CC/HTNW and CC/NW-450 °C. The black carbon cloth absorbs light in the wavelength range of 250–800 nm, and the UV adsorption edge can be seen clearly after TiO_2_ precipitations. According to the Kubelka–Munk formula, assuming an indirect transition between valence and conduction bands [24], the bandgap for the CC/NW-450 °C is evaluated to be 2.97 eV (Figure 3a inset). This value is smaller than the bandgap of 3.2 eV for anatase TiO_2_, which can be attributed to the strong interaction between TiO_2_ and the carbon cloth, which may induce localized states within the bandgap and potentially introduce defects into the TiO_2_ lattice.

Figure 3b shows the nitrogen adsorption–desorption isotherm of the TiO_2_ nanowires on carbon cloth at 77 K. The adsorption isotherm exhibits a type-IV profile with an H3 hysteresis loop, indicative of mesoporous structures with slit-like pores. This is further corroborated by the pore size distribution, which shows a sharp peak at approximately 4.0 nm, suggesting a uniform pore size. The corresponding pore size distribution curve (Figure 3b, inset) indicates a mesoporous structure. Before calcination, the BET surface area of the titanate on carbon cloth was found to be 3.9 m^2^·g^−1^ and a pore volume of 0.025 cm^3^·g^−1^, indicating limited surface area and porosity. The average pore diameter was 3.83 nm, suggesting the presence of mesopores (Supporting Information File 1, Figure S2). Note here that the carbon cloth substrate was included when evaluating the BET specific surface area. After calcination at 450 °C, the BET surface area increased remarkably to 289.9 m^2^·g^−1^, reflecting a significant enhancement in surface properties because of the activation of the carbon cloth via simple air calcination [17]. The pore volume is 0.047 cm^3^·g^−1^, with an average pore diameter of 3.82 nm.

TG analysis has been carried out, and the results are shown in Supporting Information File 1, Figure S4. The weight loss of CC/NW-450 °C is 2.16 wt %, which can be attributed to the physically adsorbed water. For CC/HTNW, 9.62 wt % weight loss is recorded, which is a result of additional water evaporation upon the decomposition of hydrogen titanate, according to Equation 4.


[4] $${\text{H}}_{2}{\text{Ti}}_{5}{\text{O}}_{11}\cdot 3{\text{H}}_{2}\text{O}\to 5{\text{TiO}}_{2}+4{\text{H}}_{2}\text{O}$$

**Figure 4:**
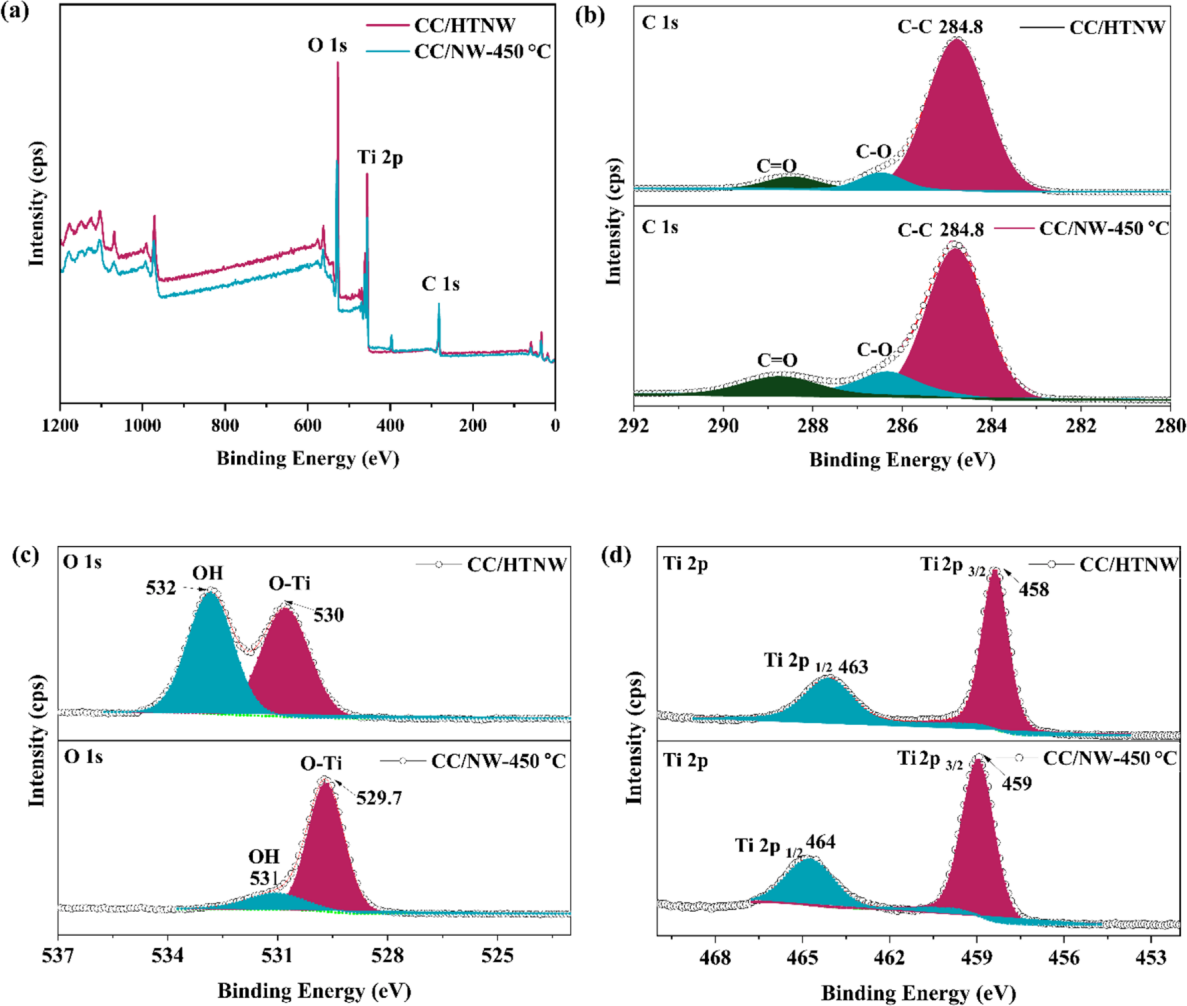
XPS spectra of CC/NW-450 °C and CC/HTNW. (a) Survey spectra and detailed XPS spectra of (b) C 1s, (c) O 1s, and (d) Ti 2p.

The surface composition and oxidation states of TiO_2_ nanowires on carbon cloth (CC/NW-450 °C) and CC/HTNW were examined by XPS (Figure 4). The survey spectrum confirms the presence of Ti, O, and C (Figure 4a) in both samples. The C 1s spectrum shows a peak at 284.8 eV, corresponding to adventitious carbon, which was used as the reference for binding energy calibration. Additional peaks at higher binding energies (286–288 eV) are attributed to oxygen-containing functional groups (C–O and C=O), indicating partial oxidation of the carbon surface. The Ti 2p spectra (Figure 4d) show characteristic peaks for CC/HTNW, with Ti 2p_3/2_ and Ti 2p_1/2_ peaks appearing at 458 and 463 eV, respectively, while for CC/NW-450 °C, these peaks slightly shift to 459 and 464 eV, respectively. The spin–orbit splitting remains ≈5.4 eV confirming the Ti^4+^ state from the lattice [25]. The O 1s spectra (Figure 4c) show significant differences between the two samples. For CC/HTNW, two peaks are observed at 530 and 532 eV, corresponding to lattice oxygen and surface hydroxyl groups (–OH), respectively. After calcination (CC/NW-450 °C), the relative intensity of the hydroxyl peak decreases, and the peaks shift slightly (O–Ti at 529.7 eV and –OH at 531 eV), indicating a reduction in surface –OH groups due to dehydration and phase transformation from hydrogen titanate to anatase TiO_2_. Based on the area underneath the corresponding O 1s XPS peak, the content of hydroxyl groups in CC/HTNW is evaluated to be ca. 52%, which reduced to approximately 16% after the final air calcination, suggesting abundant residual surface oxygen vacancies in CC/NW-450 °C. These vacancies are beneficial for enhancing photocatalytic activity [26–27].

### Photocatalytic activity for OFL removal and materials durability

**Figure 5:**
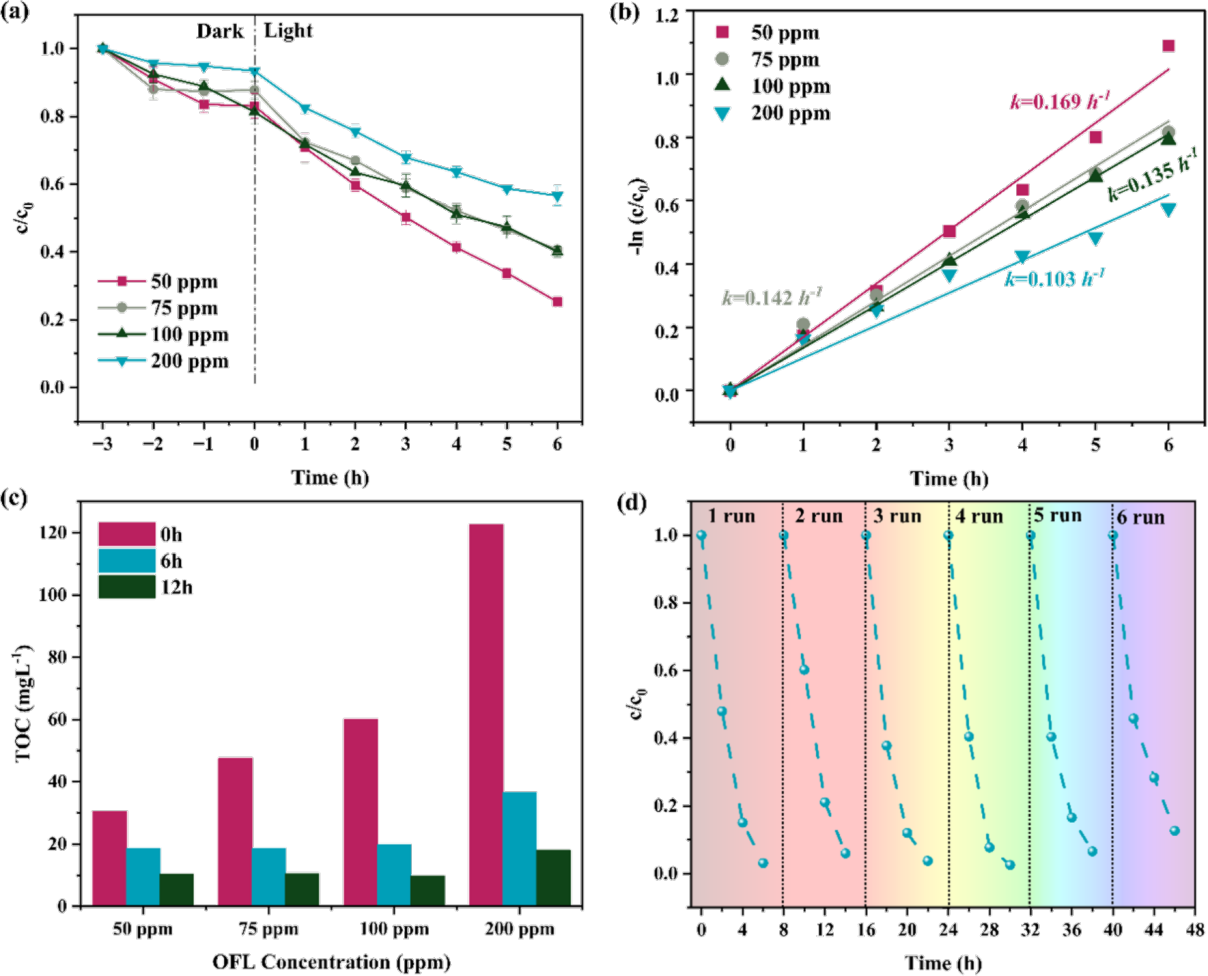
(a) Photocatalytic degradation of ofloxacin in water with various initial concentrations (50, 75, 100, and 200 ppm) assisted by CC/NW-450 °C under UV irradiation. (b) The corresponding fit curves assume pseudo-first-order reaction kinetics. (c) TOC removal after 6 and 12 h. (d) Cycling performance. Initial OFL concentration: 50 ppm.

The photocatalytic activities of the carbon cloth-supported TiO_2_ nanowires (CC/NW-450 °C) were assessed by monitoring the degradation of various concentrations of OFL aqueous solutions under UV light radiation, as shown in Figure 5a. The corresponding reaction rate constants are shown in Figure 5b. Not surprisingly, the photocatalytic activity decreases with increasing OFL initial concentration (50, 75, 100, and 200 ppm), with the reaction rate constant declining (0.169, 0.142, 0.135, and 0.103 h^−1^, respectively). The high photocatalytic performance at lower OFL concentrations is attributed to the more efficient utilization of active sites and better light penetration in the solution. The TOC removal efficiency of the carbon cloth-supported TiO_2_ nanowires (CC/NW-450 °C) was also evaluated, as shown in Figure 5c. The TOC values decreased over time for all tested concentrations of OFL (50–200 ppm), indicating progressive mineralization of the pollutant. At 50 ppm, the TOC decreased from 51.04 to 17.31 mg·L^−1^ after 12 h, corresponding to a 66% reduction. At 200 ppm, the TOC decreased from 205.1 to 30.25 mg·L^−1^ (12 h), achieving an 85% reduction, which demonstrates effective mineralization even at high pollutant loads. This trend aligns with the photocatalytic degradation kinetics (Figure 5b), where the reaction rate constant was the highest at lower concentrations (0.169 h^−1^ for 50 ppm vs 0.103 h^−1^ for 200 ppm). While the degradation rate was higher at lower concentrations due to efficient light penetration and active site utilization, the absolute TOC removal was significantly higher at 200 ppm, highlighting the catalyst’s capacity to mineralize large organic loads under optimized conditions.

The reusability of the CC/NW-450 °C photocatalyst was examined through repetitive degradations of 50 ppm OFL solution under UV light irradiation (Figure 5d). Unlike the experiment in Figure 5a, which included a dark adsorption (from −3 to 0 h) allowing OFL to adsorb onto the photocatalyst before light exposure, the cycling experiments in Figure 5d were conducted under UV light without a prior dark adsorption. This difference in experimental conditions accounts for the variation in degradation profiles between the two figures. After 6 h of irradiation, approximately 96.9% OFL degradation was achieved on the first run. This suggests that dynamic adsorption and simultaneous photocatalytic degradation of target molecules contribute to a higher efficiency for pollutant removal. The photocatalytic efficiency remained consistently high throughout the next 5 runs, with degradation efficiencies between 90% and 95% after 6 h. The fact that the photocatalyst retained over 90% of its efficiency even after 6 cycles demonstrates the stability of the CC/NW-450 °C photocatalyst. SEM and XRD data of the catalyst after the recycling experiment are shown in Figure S8 in Supporting Information File 1, from which no remarkable change can be seen because of its high structural stability. High photocatalytic activity over multiple cycles, along with simple, low-cost, and stable operation, are the key parameters for their promising applications in practical wastewater treatment, in which the frequent replacement or regeneration of the catalyst must be avoided.

### Influence of the hydrogen peroxide additive

**Figure 6:**
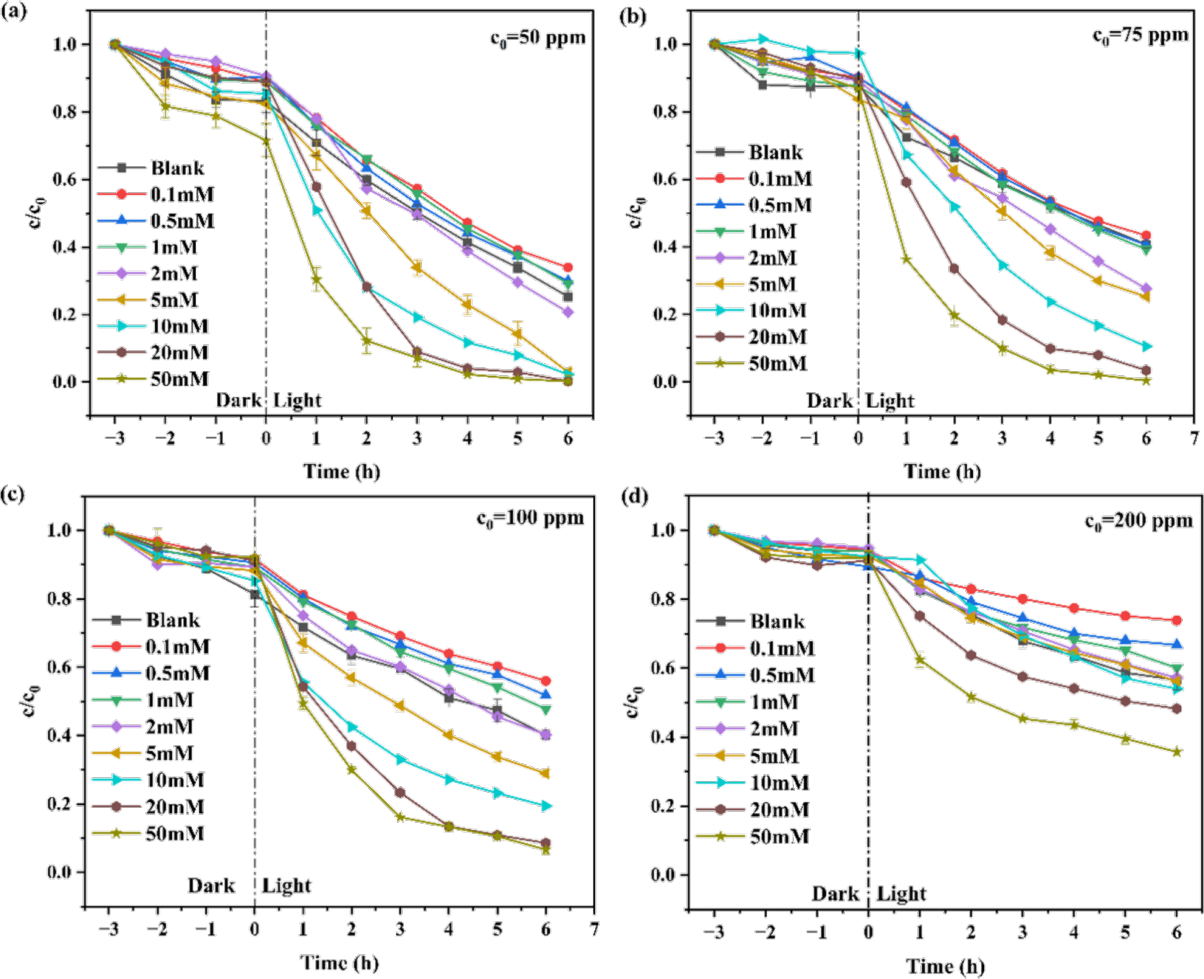
Photocatalytic degradation of various ofloxacin concentrations of (a) 50 ppm, (b) 75 ppm, (c) 100 ppm, and (d) 200 ppm in the presence of different concentrations (0.1–50 mM) of hydrogen peroxide. Note here that the error bars were provided only for selected curves.

**Figure 7:**
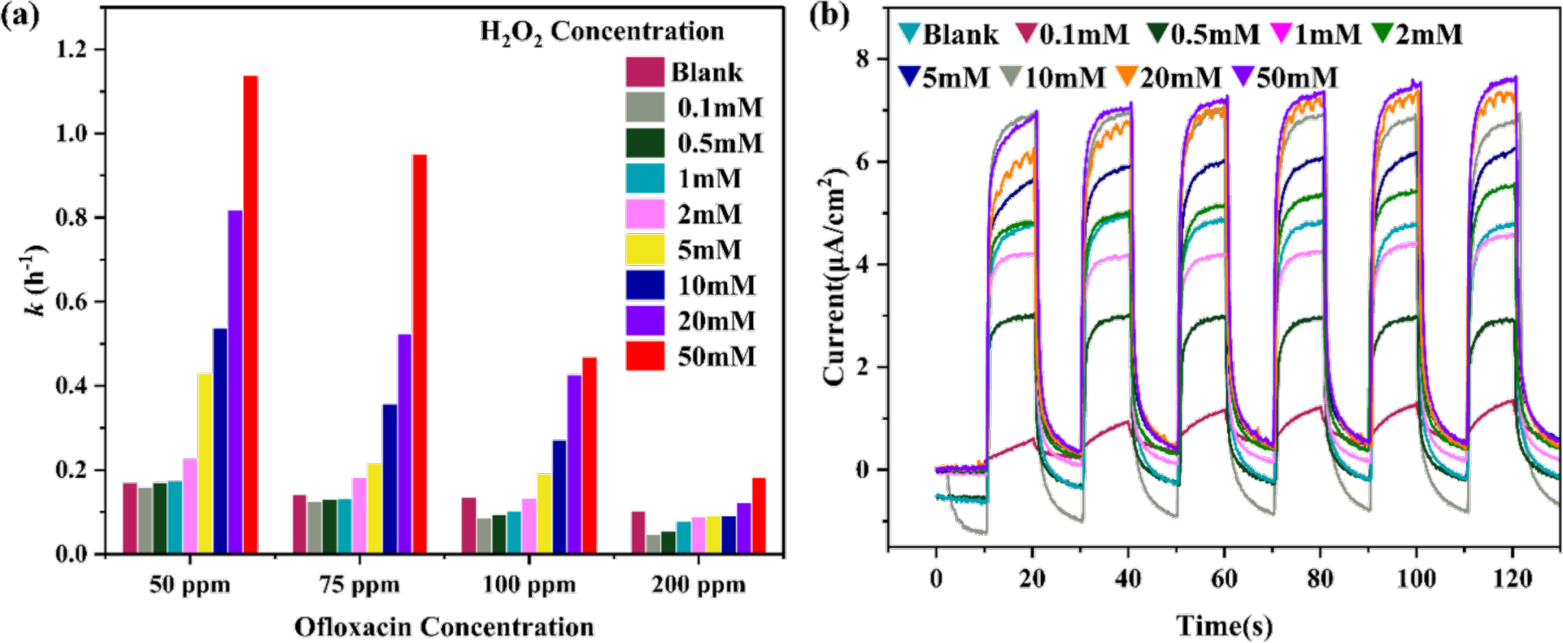
(a) Reaction rate constants of ofloxacin degradations assisted with CC/NW-450 °C and in the presence of H_2_O_2_ with various concentrations. The initial ofloxacin concentrations are 50, 75, 100, and 200 ppm. (b) Transient photocurrent responses of the CC/NW-450 °C under periodic light on/off cycles in electrolytes with different H_2_O_2_ concentrations. Initial OFL concentration: 50 ppm.

Figure 6a–d presents the photocatalytic degradation curves of OFL with various concentrations of 50, 75, 100, and 200 ppm, assisted by the carbon cloth-supported TiO_2_ nanowires and in the presence of H_2_O_2_ with various concentrations. The corresponding reaction rate constants are summarized in Figure 7a. The reaction rate constants for OFL in water with initial concentrations of 50 and 75 ppm increased gradually with increasing H_2_O_2_ additives. After 6 h, the removal is approximately 85% and 76% for 50 and 75 ppm OFL, respectively, in the presence of 10 mM H_2_O_2_. More complicated phenomena are observed for OFL in water with a higher initial concentration of 100 and 200 ppm. Upon increasing the H_2_O_2_ concentration from 0.1 to 5 mM, the reaction rate constant vs H_2_O_2_ concentration curve exhibits a “U” shape, which suggests that there is a critical H_2_O_2_ concentration, only beyond which the H_2_O_2_ additive contributes to OFL degradation. This dual behavior can be attributed to competing roles of H_2_O_2_ in advanced oxidation processes. At sub-critical concentrations, H_2_O_2_ may act as a scavenger of hydroxyl radicals (OH^•^) or holes (h^+^), reducing their availability for OFL degradation, as explained by (Equation 5):


[5] $${\text{H}}_{2}{\text{O}}_{2}+{\text{OH}}^{•}\to {\text{HO}}_{2}^{\;•}+{\text{H}}_{2}\text{O (scavenging effect)}$$

Above the critical concentration, H_2_O_2_ primarily serves as an electron acceptor (Equations 6 and 7), generating additional OH^•^ radicals via reactions with superoxide radicals (O_2_^•–^) or photogenerated electrons (e^–^) [28], demonstrated by the subsequent reactions:


[6] $${\text{H}}_{2}{\text{O}}_{2}+{\text{O}}_{2}^{\;•-}\to {\text{OH}}^{•}+{\text{OH}}^{-}+{\text{O}}_{2}$$

[7] $${\text{H}}_{2}{\text{O}}_{2}+{\text{e}}^{-}\to {\text{OH}}^{•}+{\text{OH}}^{-}$$

These reactions demonstrate that H_2_O_2_ acts as an electron acceptor, leading to the formation of OH^•^; the resulting OH^•^ radicals drive OFL degradation, explaining the enhanced reaction rates at higher H_2_O_2_ concentrations [29]. The scavenging-to-promotion transition underscores the need to optimize H_2_O_2_ dosing, particularly for high pollutant loads (e.g., 100–200 ppm OFL), where excess H_2_O_2_ initially suppresses degradation until sufficient radicals are generated.

In the current investigation, we note that H_2_O_2_ beyond a critical concentration may be necessary to generate sufficient OH^•^ radicals for effective degradations of OFL with a high initial concentration, say, 100 ppm and 200 ppm. The photocurrent response in Figure 7b increases with increasing H_2_O_2_ concentrations in the 0.5 M Na_2_SO_4_ electrolyte, because H_2_O_2_ acts as an electron acceptor, facilitating charge separation and the generation of ROS, such as OH^•^ radicals, which directly contribute to the degradation process [30]. The effect of H_2_O_2_ concentration on photocatalytic OFL degradation in the presence of hydroxyl radical scavengers was investigated using *n*-butanol as a probe molecule (Supporting Information File 1, Figure S6). In these experiments, 10 mM *n*-butanol was added to serve as an •OH scavenger to evaluate the role of hydroxyl radicals in the degradation process. The results demonstrate a clear concentration-dependent effect of H_2_O_2_ on OFL removal efficiency. At low H_2_O_2_ concentrations (5 mM), OFL degradation was comparable to the catalyst alone, indicating that the limited •OH radical generation was effectively quenched by *n*-butanol. As H_2_O_2_ concentration increased to 10 mM, a slight improvement in degradation was observed, suggesting that •OH radical production began to exceed the scavenging capacity of *n*-butanol. The most significant enhancement occurred at 50 mM H_2_O_2_, where OFL degradation reached approximately 60% after 3 h, demonstrating that high H_2_O_2_ concentrations can overcome the inhibitory effect of radical scavengers through increased ROS generation. This trend confirms the dominant role of •OH radicals in the photocatalytic degradation mechanism.

Supporting Information File 1, Figure S7 explores the effect of the pH value on the OFL degradation efficiency. The highest degradation occurs at a slightly alkaline pH (pH 8), where ROS generation and pollutant adsorption onto TiO_2_ are the most favorable. Both strong acidic (pH 2) and highly basic (pH 12) conditions reduce the degradation efficiency, likely due to reduced ROS stability or altered surface interactions. It demonstrates that the carbon cloth-supported TiO_2_ nanowires are capable of functioning in wastewater with a wide pH range.

**Figure 8:**
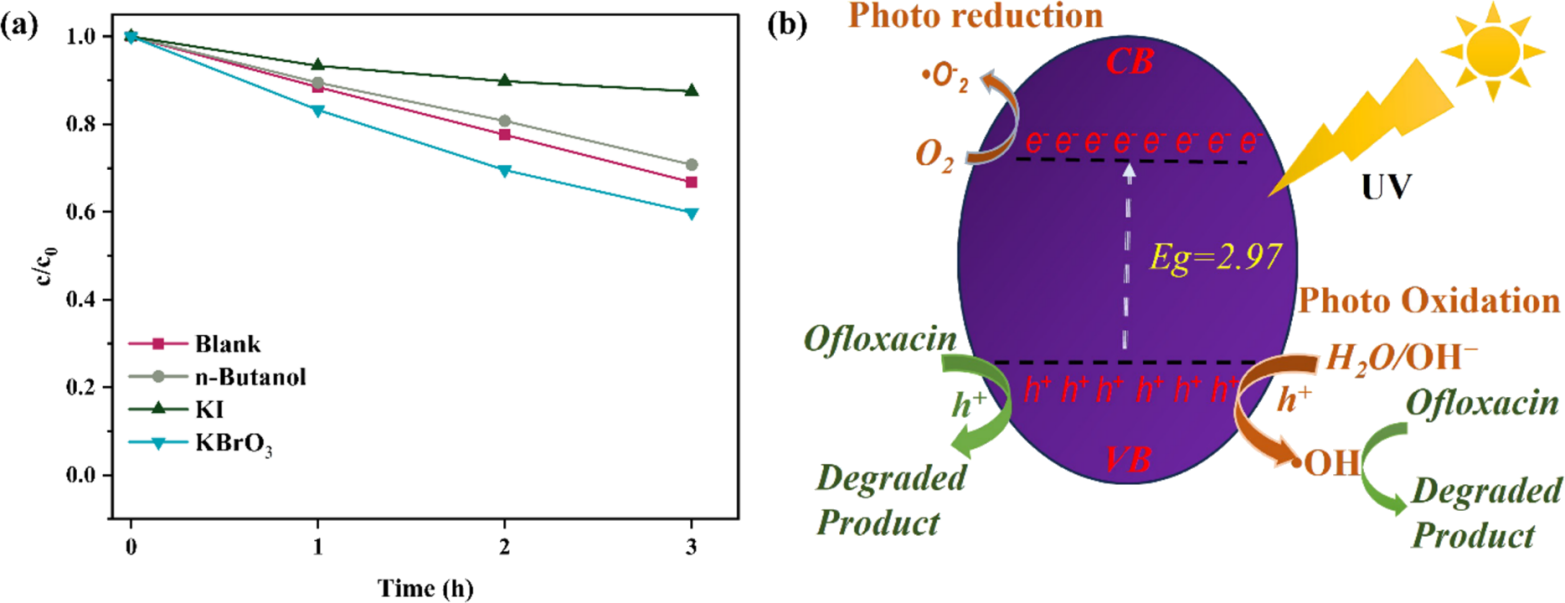
OFL degradations assisted by TiO_2_ nanowires on carbon cloth with different radical trapping agents. The initial concentration is 50 ppm. (b) Radical pathway of the photocatalytic OFL degradation in the presence of CC/NW-450 °C.

The active radical species produced during photocatalytic OFL degradations were clarified using trapping studies. *n*-Butanol, potassium iodide (KI), and potassium bromate (KBrO_3_) were employed as quenchers for hydroxyl radicals [31], holes [32], and e^−^ [33], respectively. Figure 8 illustrates that *n*-butanol reduced the OFL removal of 50 ppm to 30% over 3 h, indicating a significant supporting function of singlet hydroxyl radicals. The introduction of KI further reduced the OFL removal to 13% after 3 h of reaction, underscoring the pivotal function of holes as the principal reactive species accountable for the OFL degradation in our system. The introduction of KBrO_3_ resulted in a relatively modest reduction in the removal to 41%, suggesting that the involvement of e^−^ is minor in the process. These results indicate that the photocatalytic degradation mechanism is predominantly governed by photogenerated holes, followed by hydroxyl radicals.

TiO_2_ nanowires generate electron–hole pairs upon excitation by UV light (Equation 8). The photogenerated holes are the predominant reactive species. These holes can directly oxidize OFL molecules or water molecules adsorbed on the surface, resulting in the formation of hydroxyl radicals. Holes can oxidize hydroxide ions to generate hydroxyl radicals (Equation 9), which, although secondary, significantly contribute to the degradation process. The photogenerated electrons may react with oxygen molecules in the solution to produce superoxide radicals (Equation 9). Nonetheless, their role is insignificant in the current investigation. OFL may be compromised by those reactive species (Equations 12–14). The potential photocatalytic degradation mechanisms of OFL are thus delineated as follows and demonstrated schematically in Figure 8b.


[8] $${\text{TiO}}_{2}+h\nu \to {\text{TiO}}_{2}\left({\text{e}}^{-}+{\text{h}}^{+}\right)$$

[10] $${\text{h}}^{+}+{\text{H}}_{2}\text{O}\to {\text{OH}}^{•}+{\text{H}}^{+}$$

[9] $${\text{h}}^{+}+{\text{OH}}^{-}\to {\text{OH}}^{•}$$

[11] $${\text{e}}^{-}+{\text{O}}_{2}\to {\text{O}}_{2}^{\;•-}$$

[12] $$\text{OFL}+{\text{h}}^{+}\to \text{degradation product }\left(\text{major}\right)$$

[13] $$\text{OFL}+{\text{OH}}^{•}\to \text{degradation products }\left(\text{major}\right)$$

[14] $$\text{OFL}+{\text{e}}^{-}\to \text{degradation product}$$

### The effects of the substrate with high adsorption capability

**Figure 9:**
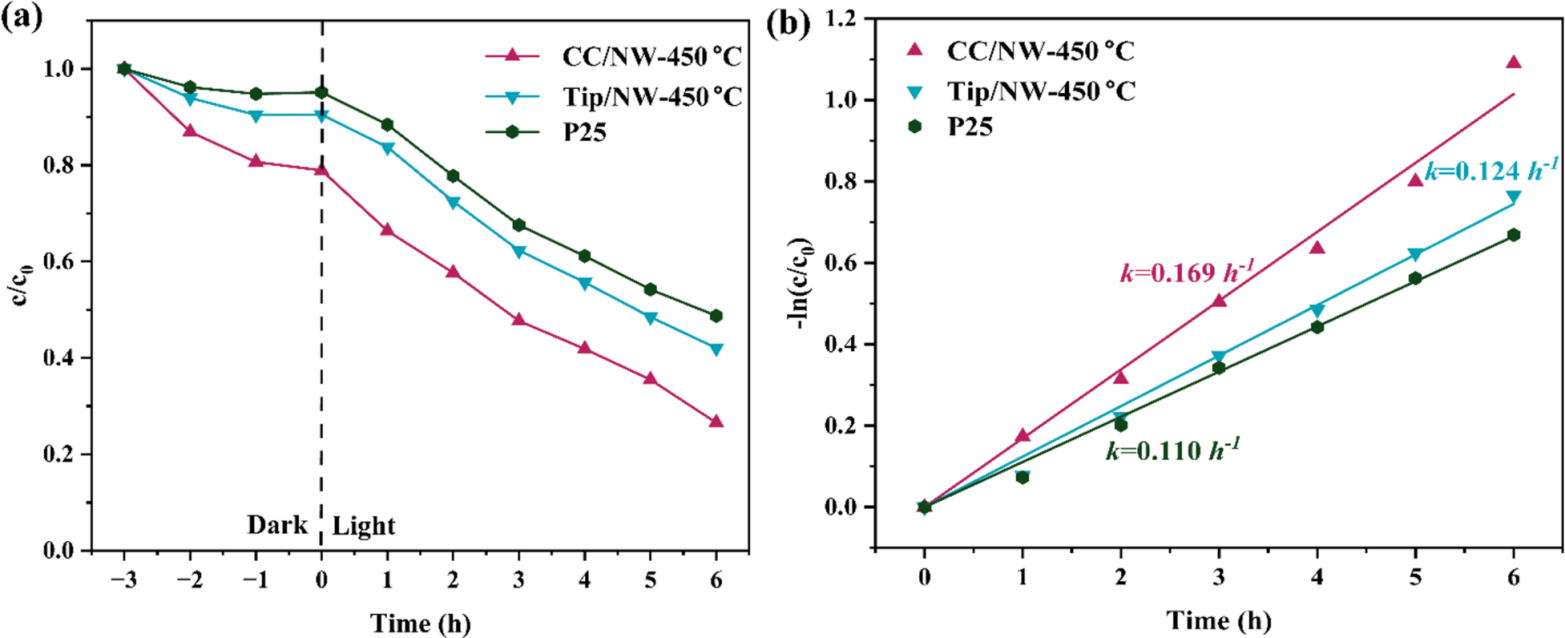
(a) Photocatalytic degradation curves of 50 ppm ofloxacin assisted by TiO_2_ nanowires on carbon cloth (CC/NW-450 °C), TiO_2_ nanowires on Ti plate (Tip/NW-450 °C), and the benchmark P25 on Ti plate (P25). (b) The corresponding fit results assuming pseudo-first-order reaction kinetics.

The contact angle measurements in Supporting Information File 1, Figure S9 reveal distinct differences in surface wettability among the samples. The pristine carbon cloth shows a high contact angle of approximately 138.9°, indicating strong hydrophobicity (Supporting Information File 1, Figure S9b). Remarkably, the CC/NW-450 °C composite exhibits a contact angle of 0° (Supporting Information File 1, Figure S9c), showing complete wettability. This significant enhancement in hydrophilicity is attributed to the high surface roughness and porous hydrophilic nanowire architecture formed on the carbon cloth, which improves water spreading and facilitates the adsorption of aqueous pollutants during photocatalytic degradation [34]. The Ti foil covered with TiO_2_ nanowires (TiP/NW-450 °C) exhibits a contact angle of around 62.4° (Supporting Information File 1, Figure S9a), reflecting a moderate hydrophilicity. Figure 9a shows the photocatalytic degradation curves and the corresponding fit results assuming a pseudo-first-order reaction for OFL with an initial concentration of 50 ppm and in the presence of TiO_2_ nanowires quasi-aligned on carbon cloth and metallic Ti substrates. The efficiency is also compared with the benchmark commercial P25 TiO_2_ nanoparticles deposited on a Ti substrate. All thin films were controlled to be of almost identical film thickness for a reasonable comparison. Both nanowire samples exhibit a photoactivity superior to that of the benchmark P25 when utilized in the form of thin films. It also indicates that the highly adsorptive carbon cloth substrate, as suggested by the dark adsorption curves in Figure 9b, contributes to the photoactivity of TiO_2_ nanowires. This fact indicates a synergistic effect resulting from substrate adsorption and TiO_2_ photodegradation. The synergistic effect may involve (1) carbon cloth possessing a substantial specific surface area and surface mesopores, which offers numerous adsorption sites, thereby concentrating solute molecules near the surface of thin films [13], (2) the transfer of photogenerated electrons from TiO_2_ to the conductive carbon cloth, facilitating the separation of photogenerated electron–hole pairs [13,35], and (3) the substrate’s fibrous structure significantly enhancing the effective contact area and preventing the aggregation of nanowires.

## Conclusion

TiO_2_ nanowire thin films of ca. 1.5 μm in thickness were precipitated on carbon cloth via Ti–H_2_O_2_ interaction, followed by a subsequent calcination in air. The synthetic process also activated the carbon cloth, significantly increasing the specific surface area to 289.9 m^2^·g^−1^. The TiO_2_ nanowires supported on carbon cloth demonstrated excellent efficiency for degradation of ofloxacin in water with high initial concentrations of 50–200 ppm. Importantly, introducing H_2_O_2_ beyond a critical concentration further improved degradation efficiency by facilitating generation of reactive oxygen species, especially hydroxyl radicals. Trapping experiments suggest that the photogenerated holes were the primary factor in the photocatalytic mechanism, while the cooperative effects of hydroxyl radicals facilitated the process. A scavenging-to-promoting transition was thus noted concerning the effects of the H_2_O_2_ additives. The TiO_2_ nanowire arrays on carbon cloth exhibited an improved photoactivity compared to the counterparts on metallic Ti foils, which can be attributed to a synergetic effect resulting from a greatly increased adsorption capacity and the considerable photodegradation efficiency by TiO_2_ nanowires. Significant TOC removal (85%) was recorded for ofloxacin in water with a high concentration of 200 ppm, suggesting the high adsorption capacity and probably a deep mineralization capacity of the thin film photocatalyst developed in the current investigation. This work represents a new promising strategy to tackle emerging contaminants and could lead to the advancement of cutting-edge materials for environmental remediation.

## Supporting Information

File 1 Additional figures.

## Data Availability

Data generated and analyzed during this study is available from the corresponding author upon reasonable request.
