# Supplementary material for: Photocatalytic degradation of ofloxacin in water assisted by TiO2 nanowires on carbon cloth: contributions of H2O2 addition and substrate absorbability
Source: Beilstein J Nanotechnol. 2025 Sep 8;16:1567–79. doi: 10.3762/bjnano.16.111 (PMC12434924; doi:10.3762/bjnano.16.111)
Supplement: File 1 — Additional figures. [file Beilstein_J_Nanotechnol-16-1567-s001.pdf]

BEILSTEIN JOURNAL OF NANOTECHNOLOGY

Supporting Information

for

Photocatalytic degradation of ofloxacin in water assisted by TiO_2 nanowires on carbon cloth: contributions of H_2O_2 addition and substrate absorbability

Iram Hussain, Lisha Zhang, Zhizhen Ye and Jin-Ming Wu

Beilstein J. Nanotechnol. **2025,** *16,* 1567–1579. doi:10.3762/bjnano.16.111

Additional figures

License and Terms: This is a supporting information file under the terms of the Creative Commons Attribution License (https://creativecommons.org/licenses/by/4.0). Please note that the reuse, redistribution and reproduction in particular requires that the author(s) and source are credited and that individual graphics may be subject to special legal provisions.
The license is subject to the *Beilstein Journal of Nanotechnology* terms and conditions: (https://www.beilstein-journals.org/bjnano/terms)

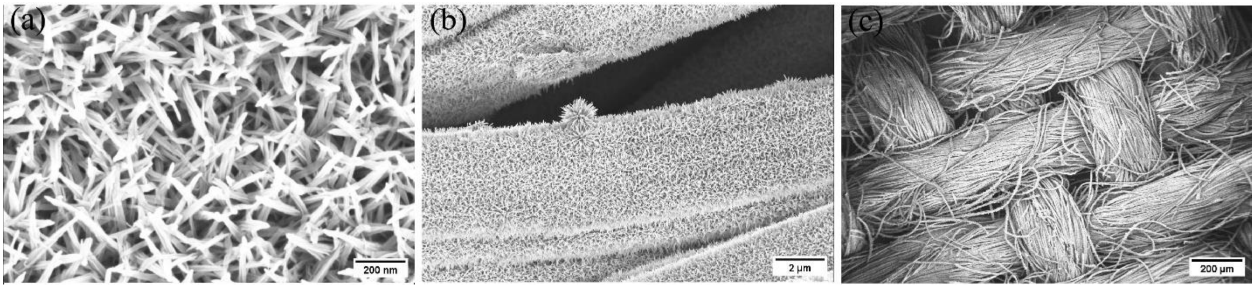

Figure S1: SEM images of hydrogen titanate nanowires grown on carbon cloth at different magnifications: (a) High-magnification image showing the dense network of hydrogen titanate nanowires, (b) intermediate magnification highlighting the uniform coating of nanowires on the individual carbon fibers, and (c) low-magnification image displaying the woven structure of the carbon cloth substrate.

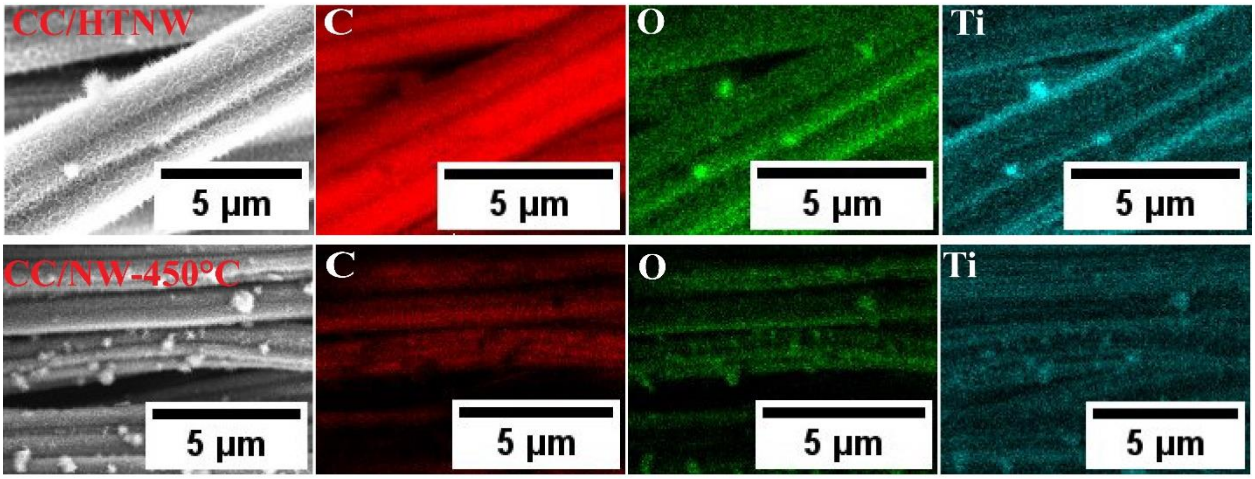

Figure S2: EDX elemental mapping images showing the distribution of Ti and O on the carbon cloth substrate coated with hydrogen titanate nanowires (CC/HTNW) and TiO_2 nanowires (CC/NW-450 °C).

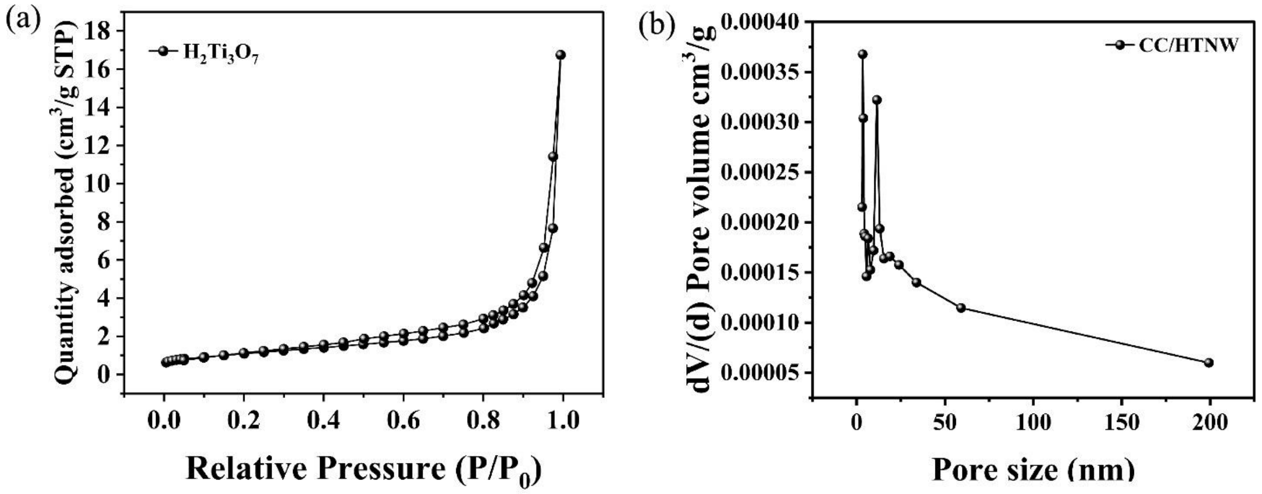

Figure S3: (a) Nitrogen adsorption-desorption isotherm for the carbon cloth substrate coated with hydrogen titanate nanowires (CC/HTNW), showing the quantity adsorbed (cm^3/g STP) as a function of relative pressure (P/P_0). (b) Pore size distribution derived from the adsorption data, plotted as the derivative of pore volume ($dV/d\log(D)$) versus pore diameter.

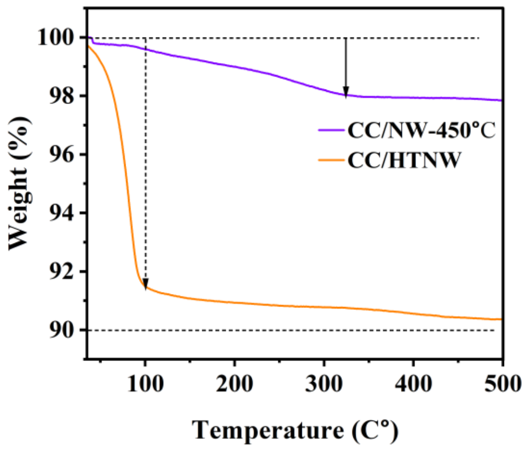

Figure S4: Thermogravimetric analysis (TGA) of hydrogen titanate (CC/HTNW) and the TiO_2 nanowires calcined at 450 °C (CC/NW-450 °C), precipitated on carbon cloth.

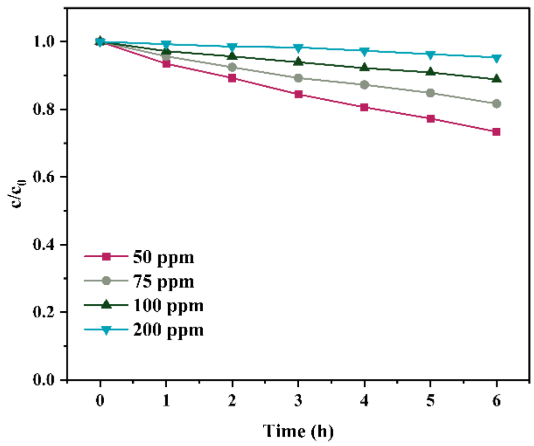

Figure S5: Dark adsorption of ofloxacin at different initial concentrations (50, 75, 100, and 200 ppm) in the presence of CC/NW-450 °C composite over 6 h. The plot shows the change in normalized concentration (c/c_0) with time, illustrating the adsorption capacity of the TiO_2 nanowire-coated carbon cloth before illumination.

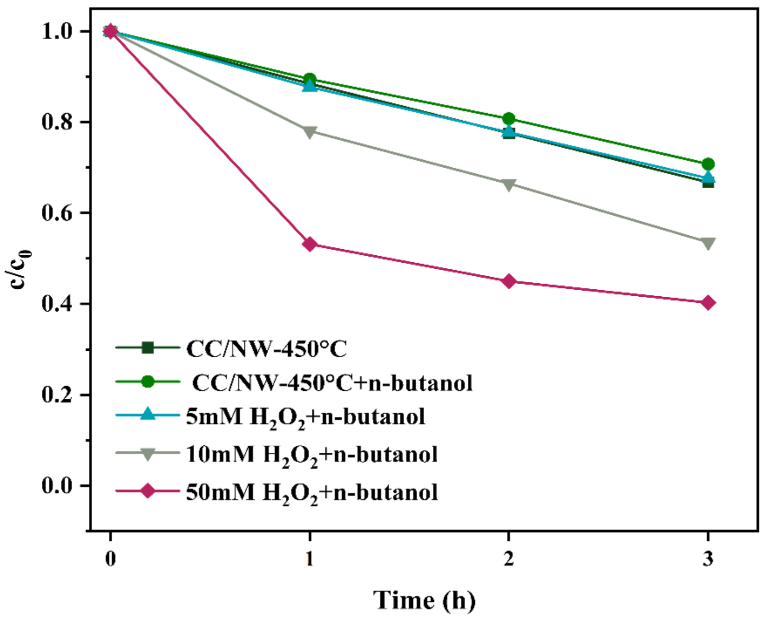

Figure S6: Normalized concentration (c/c_0) of ofloxacin over time in the presence of the CC/NW-450 °C and varying H_2O_2 concentrations, monitored with *n*-butanol as a probe for reactive oxygen species. Initial OFL concentration: 100 ppm.

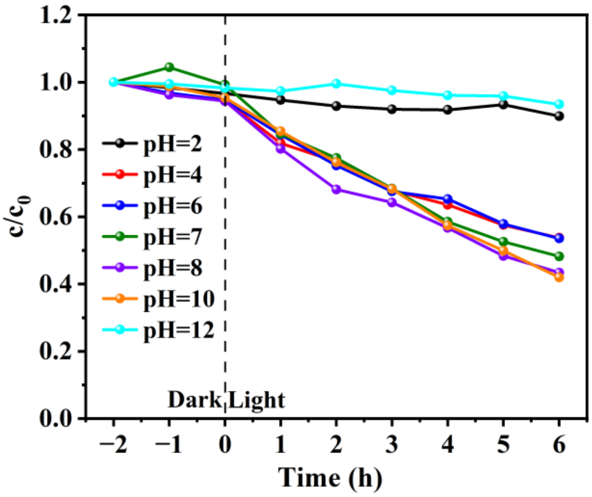

Figure S7: Photocatalytic degradation of ofloxacin (100 ppm) using CC/NW-450 °C under varying pH conditions (pH 2–12). The catalyst works in a wide pH range of 4–10.

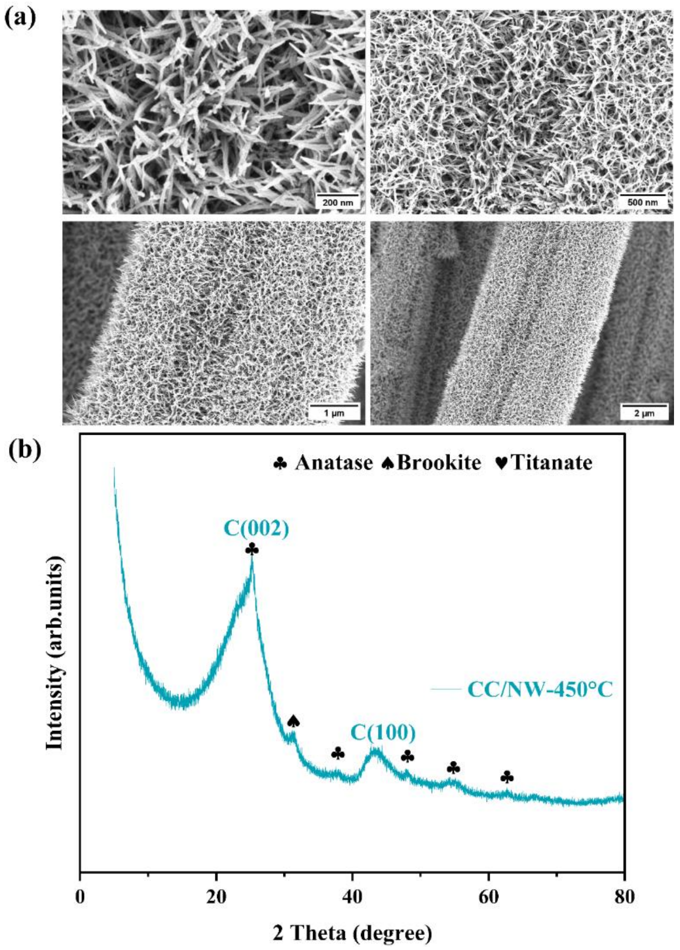

Figure S8: (a) FESEM and (b) XRD pattern of CC/NW-450 °C after 6th repeated use, suggesting the morphological and structural stability.

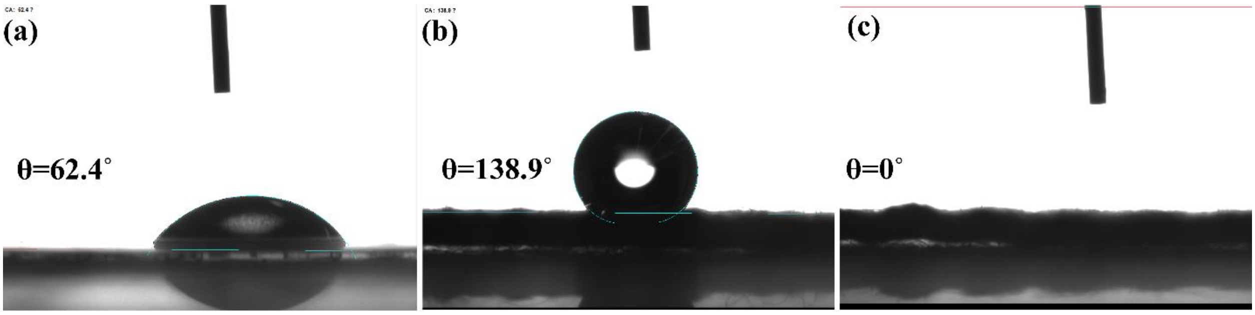

Figure S9: Contact angle images showing surface wettability of different samples: (a) Tip/NW-450 °C (θ = 62.4°), (b) pristine carbon cloth (CC) (θ = 138.9°), and (c) CC/NW-450 °C (θ = 0°).
